# Effects of strength training on functional ambulation following knee replacement: a systematic review, meta-analysis, and meta-regression

**DOI:** 10.1038/s41598-023-37924-1

**Published:** 2023-07-03

**Authors:** Jinxiang Wang, Ranran Zhu, Xiao-ting Xu, Shuting Liu, Zhenrui Li, Chaoyang Guo, Xuchen Tao, Liang Qian, Ravon Charles, Lei Fang

**Affiliations:** 1 Yueyang Hospital of Integrated Traditional Chinese and Western Medicine, Shanghai University of Traditional Chinese Medicine, Shanghai, China; 2 School of Rehabilitation Science, Shanghai University of Traditional Chinese Medicine, Shanghai, China; 3 Institute of TCM International Standardization, Shuguang Hospital Affiliated to Shanghai University of Traditional Chinese Medicine, Shanghai, China

**Keywords:** Diseases, Medical research

## Abstract

Strength training is recommended by the American Physical Therapy Association to improve muscle strength, mobility, and balance following knee replacement. Few studies have focused on the direct effects of strength training on functional ambulation, and potential dose–response relationships between strength training parameters and the effect remain unclear. The aim of this systematic review, meta-analysis, and meta-regression was to evaluate the effects of strength training on functional ambulation following knee replacement (KR). We also aimed to explore potential dose–response relationships between strength training parameters and performance in functional ambulation. A systematic literature search of eight online databases was performed on March 12, 2023, for randomized controlled trials evaluating the effects of strength training on functional ambulation by six-minute walk test (6MWT) or timed-up and go test (TUG) after KR. Data were pooled by random-effect meta-analyses and presented as weighted mean difference (WMD). A random-effect meta-regression was performed for four predetermined training parameters, namely, duration (weeks), frequency (sessions per week), volume (time per session), and initial time (after surgery) separately to explore dose–response relationships with WMD. Fourteen trials encompassing 956 participants were included in our study. Meta-analyses showed an improvement in 6MWT performance after strength training (WMD: 32.15, 95% CI 19.44–44.85) and a decrease in time to complete TUG (WMD: − 1.92, 95% CI − 3.43 to − 0.41). Meta-regression revealed a dose–response relationship only between volume and 6MWT, with a decreasing trend (*P* = 0.019, 95% CI − 1.63 to − 0.20). Increasing trends of improvement in 6MWT and TUG were observed with increasing training duration and frequency. A slight decreasing trend of improvement was observed in 6MWT with postponed initial time, while an opposite trend was observed in TUG. Based on existing studies, moderate-certainty evidence suggests that strength training could increase 6MWT distance, and low-certainty evidence shows that strength training could decrease the time to complete TUG after KR. Meta-regression results only suggested a dose–response relationship between volume and 6MWT with a decreasing trend.

Registration: PROSPERO: CRD42022329006.

## Introduction

Knee osteoarthritis (KOA) is a considerable global health burden that may cause knee pain, stiffness, high risk of falls, and even disability^1,2^, the prevalence of which has doubled since the mid-twentieth century with the global prevalence of radiographically confirmed KOA being 3.8% in 2010^2,3^. Pharmacological therapies, physical therapies, and joint-preserving surgical treatment might slow down the progression of the disease and alleviate pain before end-stage KOA^4^. If these treatments fail, with consistent pain and dysfunction, knee replacement (KR) could be the final choice^4,5^. It has been reported that 80–85% of patients are satisfied after surgery, but there is a small proportion of patients who experience postsurgi pain and functional ambulation limitations due to muscle impairment, weakness, and neuromuscular activation deficits^5,6^.

Strength training is effective for attenuating muscle weakness and improving physical performance^7^. Several systematic reviews and meta-analyses have shown safety and non-inferiority in functional ambulation of strength training compared with other interventions after KR^8,9^. Moreover, strength training is strongly recommended for improving muscle strength, mobility, balance, and knee extension after KR by the American Physical Therapy Association (APTA)^10^. Despite the many benefits of strength training for patients after KR, there is still a lack of generalized parameters in clinical practice^10^, and training protocols among relevant studies vary in duration, frequency, initial time, and intensity.

We conducted the present systematic review, meta-analysis, and meta-regression focusing on functional ambulation in patients following KR. The aims of this study were as follows: (1) to evaluate the effects of strength training on functional ambulation following KR and (2) to explore the dose–response relationship between strength training parameters and functional ambulation.

## Methods

### Registration

The review was conducted in accordance with the Preferred Reporting Items for Systematic Reviews and Meta-analysis (PRISMA) checklist^11^. The review protocol was registered on the PROSPERO database (CRD42022329006).

### Search strategy

We performed a systematic literature search in April of 2022 and an updated literature search on March 12, 2023, in the following online electronic databases: PubMed, Web of Science, The Cochrane Library, Embase, Ovid, China National Knowledge Infrastructure (CNKI), Wanfang Data, and Chongqing VIP. The search strategy included the combination of keywords such as “knee replacement,” “knee arthroplasty,” “knee prosthesis,” “strength training,” “resistance training,” “weight-bearing exercise,” “eccentric exercise,” “concentric exercise,” and “isotonic exercise.” The full search strategy is available in Supplementary Table S1 online. A forward and backward search of reference lists in the included studies was performed manually.

### Eligibility criteria

The eligibility criteria were as follows.

*Participants*: The patients underwent primary KR due to KOA. Patients with severe comorbidities (such as uncontrolled diabetes and severe cardiovascular or neurological diseases) that could affect functional ambulation performance were excluded.

*Intervention*: The experimental group received strength training with or without standard care. Strength training was defined as an exercise for increasing muscle power against a force or gravity^12^. A treatment protocol or an added protocol for the intervention group was regarded as a strength training protocol if: (1) the time for strength training constituted more than half of the total training time (the time for warm-up and cool-down exercises was not calculated) and (2) the researchers hypothesized that it could cause larger improvement in muscle strength or functional ambulation than the control treatment, or else augment the improvement of muscle strength or functional ambulation on the basis of the control treatment.

*Control*: The control group received standard care or no intervention. As the APTA guideline highlights that strength training is beneficial for mobility, balance, and knee extension after KR, and due to ethical concerns, strength training is sometimes included in standard care protocols^10^. In this study, we distinguished standard care protocols from strength training protocols if: (1) strength training was one of the applied therapies (e.g., knee range of motion training, balance training, cryotherapy) in a protocol, (2) the time for strength training constituted no more than half of the total training time, and (3) the aim of the protocol was not to improve muscle strength or functional ambulation specifically. Studies of strength training in both treatment and control groups with different parameters (high intensity vs. low intensity, weight-bearing vs. non-weight-bearing, etc.) were excluded.

*Outcome*: The 6-min walk test (6MWT) was selected as the primary outcome as it reflects a person’s walking distance in 6 min, which is close to situations of daily living. The timed-up and go (TUG) test was selected as the secondary outcome. In this test, participants need to stand up from a seated position, walk 3 m, turn back, and sit down. A decrease in the time needed to complete TUG indicates an improvement in the outcome^13^.

*Study*: Randomized controlled trials (RCTs) published in English or Chinese were included, with no restrictions in terms of year, region, and ethnicity.

### Selection process

The retrieved articles were imported into Endnote 20.3, and duplications were removed. Two independent reviewers read titles and abstracts of all articles, and then a full-text evaluation of potentially eligible articles was performed. The final selection results were cross-checked between the two reviewers, and conflicts were solved by discussion or by consulting a third reviewer.

### Data extraction

Information from the eligible studies was extracted by two independent reviewers using a predesigned data extraction form. The following information was collected: (1) study characteristics: publishing year, country or region, first author, and number of participants in each group; (2) patients’ baseline characteristics: mean age, body mass index (BMI), and proportion of male/female; (3) strength training characteristics: details of training procedures, duration (weeks), frequency (sessions per week), volume (minutes per session), and initial time (after surgery); (4) outcomes of functional ambulation. Discrepancies were solved by discussion.

### Risk of bias assessment

Risk of bias was assessed by the Cochrane risk of bias assessment tool 2 (RoB2)^14^. The RoB2 tool consists of five domains: bias arising from the randomization process, bias due to deviations from intended interventions, bias due to missing outcome data, bias in the measurement of the outcome, and bias in the selection of the reported result. There are a series of signal questions for each domain, with an algorithm that maps the answers “yes,” “probably yes,” “probably no,” “no,” or “no information” to the risk of bias judgements “low risk,” “some concerns,” or “high risk.” Risk of bias for a study is rated as “low risk,” “some concerns,” and “high risk” if all domains are “low risk,” at least one domain is “some concerns” but there are no “high risk” domains, and at least one domain is “high risk” or multiple domains are “some concerns”, respectively. Two reviewers assessed the risk of bias independently, and the final results were cross-checked. Conflicts were solved by discussion or by consulting the third reviewer.

### Meta-analyses

We quantified the effects of strength training on functional ambulation by meta-analyses using the random-effect model. Results of continuous variables were expressed as weighted between-group mean difference (WMD) with a 95% confidence interval (CI). For studies with two intervention groups, we divided the control group into two (WMD remained unchanged, while the sample size and the standard deviation halved)^15^.* P* < 0.05 was considered statistically significant. In addition, we also computed the weighted mean differences within the strength training group from baseline to posttreatment by using random-effect meta-analyses. Inter-study heterogeneity was evaluated by the I^2^ statistic test. I^2^ values of 20, 50, and 75% indicate that there might be low, moderate, and high heterogeneity, respectively^16^. Publication bias was evaluated via funnel plots if there were 10 or more studies in one meta-analysis. Egger regression test was performed when the funnel plot showed obvious asymmetry, with a *P* value > 0.1 indicating potential publication bias^17^. Sensitivity analysis was performed by the leave-one-out method to find any influential studies.

### Confidence level assessment of meta-analysis results

We used the GRADE tool to evaluate the confidence level of our results in GRADEpro software (version 3.6.1). The results of meta-analyses from RCTs had a high confidence level initially. If there was risk of bias, inconsistency, indirectness, imprecision, and publication bias in the included studies, the confidence level was downgraded to moderate, low, or very low^18^.

### Meta-regression

We determined the following critical strength training parameters that might affect the training effects a priori based on the previous studies: duration (training weeks)^19^, frequency (sessions per week)^19,20^, volume (minutes per session)^21^, and initial time (days after surgery)^22^. For each outcome, random-effect univariate meta-regressions were performed by residual restricted maximum likelihood method to measure the between-study variance^23^. The dependent variable was WMD, while the parameters were selected as covariates. Multivariate meta-regression with Knapp–Hartung modification was performed if the number of parameters with statistical significance (*P* < 0.05) was more than one. Permutation tests were performed to deal with multiple testing (n = 1000)^23^. *P* < 0.05 in the multivariate meta-regression indicated a more convincing dose–response relationship between a parameter and the outcome than that in the univariate meta-regression. The bubble plot was presented for the trend of dose–response relationship. All statistical analyses and meta-regression were performed in Stata 17.0 software (StataCorp LLC, College Station, USA).

## Results

### Search results

**Figure 1:**
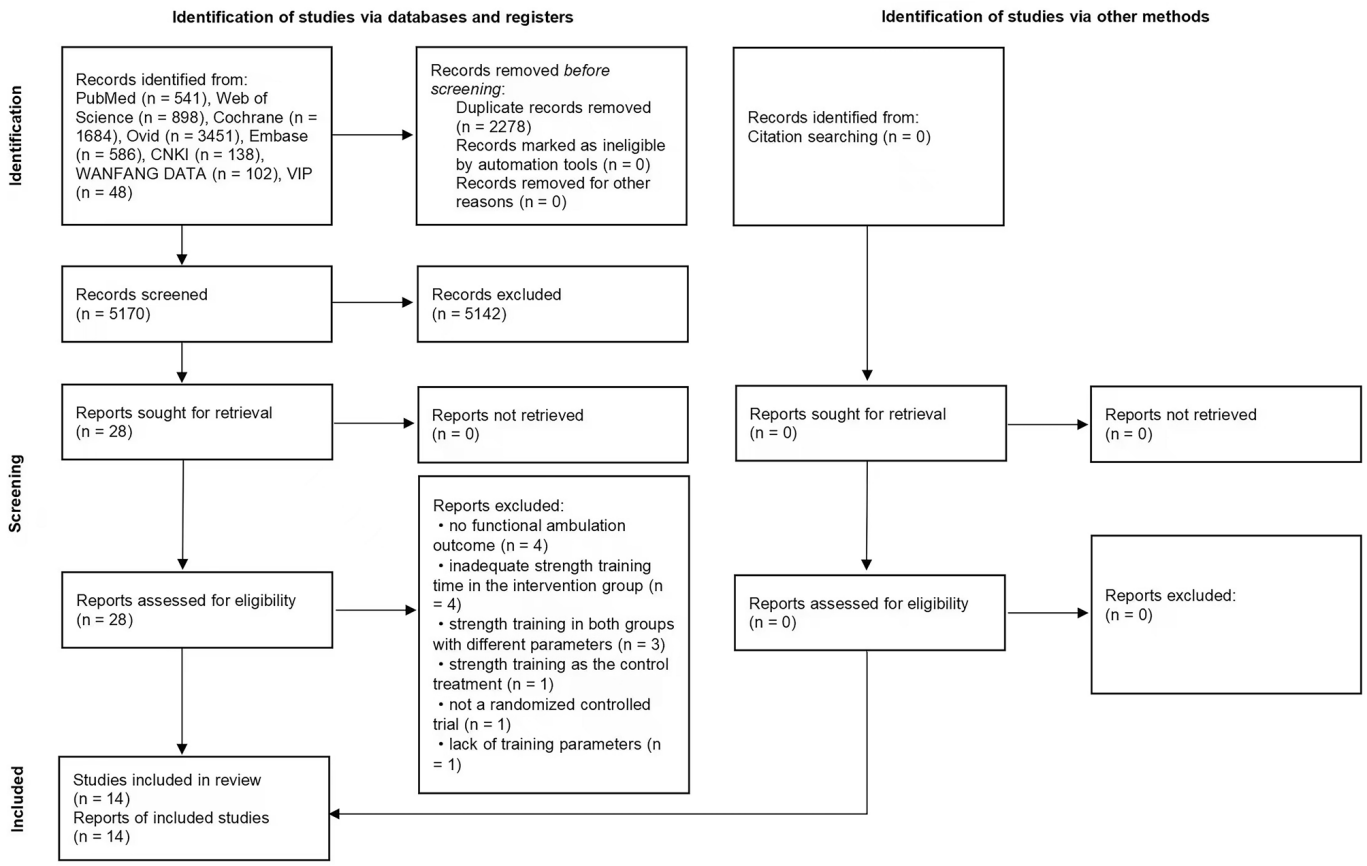
Study flow diagram.

A total of 7448 articles were retrieved, and 5170 articles remained after removing duplicates. Twenty-eight studies were full-text evaluated after title and abstract screening. Finally, 14 studies were included in meta-analyses. Fourteen studies were excluded due to the following reasons: (1) authors did not assess participants’ functional ambulation^24–27^; (2) the intervention group received more functional training (e.g., aerobic exercise and balance training) than strength training^28–31^; (3) both groups received strength training, only with different parameters^32–34^; (4) strength training was applied in the control group, and the intervention group received other treatments^35^; (5) other reasons, including non-RCT study design or a lack of training parameters^36,37^ (Fig. 1).

### Characteristics of the included studies

Publication countries of the 14 RCTs included in our study were Austria (1 study), Korea (1 study), China (4 studies), Norway (1 study), India (1 study), Denmark (2 studies), Canada (1 study), United States of America (1 study), Australia (1 study), and Finland (1 study). A total of 956 participants were included, with the mean age ranging from 61.8 to 73.1 years, and mean BMI ranging from 23.8 to 37.6 kg/m^2^.

As for the strength training protocols, five studies applied knee extension–flexion resistance training^38–42^; six studies applied strength training targeting lower limb muscles involving hip, knee, and ankle^15,29,43–46^; while another three studies targeted hip muscles^47–49^. The strength training parameters ranged from 4 to 13 (weeks) in terms of duration, from 2 to 7 in terms of frequency (times), from 15 to 70 in terms of volume (minutes), and from 2 to 423 in terms of initial time (days) (Table 1).

**Table 1:** Characteristics of the included studies.

Study	Baseline characteristics		Number of participants	Interventions		Outcome(s)	Strength training parameters			
	EG (mean, SD)	CG (mean, SD)		EG	CG		Duration (weeks):	Frequency (times per week):	Volume (minutes per session):	Initiated time (days after surgery):
Moffet 2004	Men/women: 14/24 Age: 66.7 (8.7) BMI: /	Men/women: 17/22 Age: 68.7 (8.3) BMI: /	EG: 38 CG: 39	Warm-up and stretching exercises Specific strengthening exercises: 1. Isometric for knee extensors: flex 0° 2. Isometric for knee extensors: flex 60° 3. Isometric for hamstrings: flex 60° 4. Concentric-eccentric for hip abductors Functional task-oriented exercises: 1. Getting up and sitting down 2. Knee extensor strengthening while standing with Theraband 3. Controlled bilateral knee flexion–extension while standing 4. Unilateral knee flexion to 90° while standing 5. Climbing on a platform or a flight of stairs 6. Walking backward on a slope and/or laterally while crossing lower limbs 7. Walking in place with a large amplitude of hip and knee flexion and upper-limb movements Endurance exercises Cool down	Standard care: simple exercises to retrain lower-limb strength (quadriceps, hamstrings, hip abductors and extensors) and to increase knee mobility, as well as some advice about knee positioning, ice application, and gait retraining	6MWT	8	1.5	52.5	60
Jakobsen (2014) Denmark	Men/women: 14/21 Age: 66 (56–73) BMI: 29.8 (27.5–36.0) Presented as median (IQR)	Men/women: 16/21 age: 63 (57–68) BMI: 29.4 (26.9–32.0) Presented as Median (IQR)	EG: 35 CG: 37	CG + knee extension and leg press with increased loads and decreased repetitions for progression	Standard care: warm up, range of motion exercise, stretching exercise, functional training, balance training and icing	6MWT	7	2	42.5	2
Vuorenmaa 2014 Finland	Men/women: 23/30 Age: 69 (8) BMI: 31 (5)	Men/women: 19/36 Age: 69 (9) BMI: 31 (6)	EG: 53 CG: 55	Isometric strengthening exercises for the quadricep and hamstring muscles at multiple knee joint angles, rising on the toes, stepping up and down, squatting, and hack squatting exercises	Usual care: active and passive knee range of motion exercises, knee flexor and extensor exercises, hip abductor and extensor exercises in the standing position (using the weight of an extremity as resistance) and icing	TUG	16	3	60	60
Bily 2016 Austria	Men/women: 9/17 Age: 64.9 (6.0) BMI: 28.7 (4.1)	Men/women: 9/20 Age: 68.3 (6.7) BMI: 28.0 (3.8)	EG: 26 CG: 29	Strength training on a computer-controlled, linear motor-powered leg-press machine Training progressed from purely isokinetic to eccentric-concentric	Ergometer cycling, manual therapy, and soft tissue techniques to improve scar and joint mobility, ROM-exercises, isometric and dynamic strengthening exercises, and gait-retraining exercises quadriceps setting, straight leg raise, seated knee extension, etc. without resistance	TUG	6	2	15	49
Harikesavan 2017 India	Men/women: 6/4 Age: 63.3 (5.4) BMI: 26.5 (3.2)	Men/women: 4/6 Age: 62.8 (5.9) BMI: 29.8 (3.2)	EG: 10 CG: 10	CG + hip abductor strengthening exercises abduction lying on side, unilateral hip abduction performed standing (from no resistance to ankle weights), isometric hip abductor strengthening exercise, calm exercises for hip abductors, standing wall isometric hip abduction, calm exercises with resistance with TheraBand, functional exercises, and walking	Early mobilization, such as reducing pain and swelling, improving knee flexion and extension range of motion, and progressive quadriceps strengthening exercises to maximize function	6MWT TUG	12	2.5	42.5	8
Jørgensen (2017) Denmark	Men/women: 16/15 Age: 64.8 (8.3) BMI: 29.8 (4.8)	Men/women: 10/14 Age: 64.4 (8.7) BMI: 28.4 (2.8)	EG: 31 CG: 24	Warm-up, leg press and knee extension exercises in strength training machines (Technogym®, Cesena, Italy) (week 1) and knee flexion (added from week 3) Increased sets and loads for progression	Home-based exercise programme focused mainly on blood and lymph circulation and knee range of movement	6MWT	8	2	30	3
Husby (2018) Norway	Men/women: 10/11 Age: 61 (46–72) BMI: 31 (5.3) Age presented as median (IQR)	Men/women: 8/12 Age: 63 (45–73) BMI: 28.6 (5.1) Age presented as median (IQR)	EG: 21 CG: 20	Warm-up: walking or ergometer cycling; maximal strength training: leg presses and knee extensions in the operated leg only; when participants were able to perform 6RM, the load was increased by 5 kg for leg presses and by 0.5–1 kg for knee extensions	Standard rehabilitation: 2–3 physiotherapy sessions a week for 8 weeks, telephone contact by project leader once a week, writing training diary		8	3	30	8
Schache 2019	Men/women: 15/39 Age: 70 (7) BMI: 30 (6)	Men/women: 21/30 Age: 69 (7) BMI: 31 (6)	EG: 54 CG: 51	Same usual care as CG additional hip strengthening exercises: hip abduction while lying on side, prone hip extension, sideways walking, standing hip abduction, and hip hitching	Usual care: improve quadriceps strength, increase active knee flexion range of motion, improve hamstring strength, increase knee extension range of motion, and improve calf muscle strength and flexibility Additional usual care: sitting to standing, marching, and walking	6MWT TUG	11	6	60	12
Do (2020) Korea	Men/women: 3/16 (hip), 3/17 (quad) Age: 72.84 (7.03) (hip), 72.50 (4.73) (quad) BMI: 37.19 (2.59) (hip), 37.60 (3.03) (quad)	Men/women: 3/13 Age: 73.13 (no SD reported) BMI: 37.62 (2.75)	EG (hip): 20 EG (quad): 20 CG: 20	Hip: warm up, supine extension bridge with Theraband, sideway walking with Theraband, standing hip adduction with Theraband, hip external rotation with Theraband quadriceps: warm up, seated knee extension with Theraband, supine straight leg raise with theraband, quarter wall squat with Theraband	Active range of motion exercises of the knees	6MWT TUG	12	3	30	180
Huang FF 2020 China	Men/women: 15/30 Age: 69.71 (4.15) BMI: 23.95 (2.87)	Men/women: 17/28 Age: 69.62 (4.23) BMI: 23.79 (2.66)	EG: 45 CG: 45	CG + knee extension-flexion exercises initiated immediately after surgery with sand packs weighted 1.0, 2.5, 3.5 or 4.5 kg on the ankle Massage was applied after training for muscle relaxation	Non-resistance exercises on bed: straight leg raise, ankle pump, isotonic quadriceps contraction, and passive knee range of motion training; knee extension-flexion exercises against gravity initiated 1 week after surgery	6MWT	12	7	15	7
Liao CD (2020) Tai wan, China	Age: 72.22 (7.75) BMI: 28.54 (3.88) Men/women: not reported	Age: 69.79 (6.72) BMI: 27.25 (4.36)	EG: 30 CG: 30	min warm-up, 40-min period of elastic resistance exercises, and a 10-min cool down Elastic resistance exercises: seated chest press, seated row, seated shoulder press, hip circumduction, leg press, and leg curl	Standard care: education, pharmacologic therapy, active and passive range of motion exercise, stretching exercise, and functional reconditioning exercise	TUG	12	2	60	30
Trudelle 2020 USA	Men/women: 0/6 Age: 61.8 (7.0) BMI: 33.0 (6.9)	Men/women: 2/4 Age: 66.0 (6.5) BMI: 36.7 (8.4)	EG: 6 CG: 6	CG + mini squats, step ups, sit to stand exercises; open-chained knee extension exercise with elastic band resistance; performing the concentric phase forcefully and as quickly as possible and perform the eccentric phase slowly and with control; initiated 2 years after surgery	Monitoring of daily steps by wearing a pedometer to record the number of steps; patients were encouraged to walk 7500 to 10,000 steps daily	6MWT	8	3.5	25	423
Quan HL 2022 China	Men/women: 12/18 Age: 65.27 (6.49) Weight: 65.87 (7.01) kg Height: 1.64 (0.07) m	Men/women: 17/13 Age: 66.23 (7.73) Weight: 66.07 (8.38) kg Height: 1.62 (0.07) m	EG: 30 CG: 30	CG + hip abductors resistance training while supine, lying on side, and standing; side step and side upstairs; using sand packs or elastic bands for resistance	Keeping the knee at straight position using sand packs; isotonic contraction of quadricep and hamstring muscle; ankle pump and straight leg raise without resistance 3 sets per day, 10 repetitions per set; patella mobilization; mobility training with walking aid; balance and proprioception training	TUG	4	5	40	12
Sun Q 2022 China	Men/women: 31/40 Age: 67.8 (4.05) BMI: 25.81 (2.37)	Men/women: 31/39 Age: 67.4 (3.94) BMI: 26.14 (2.46)	EG: 71 CG: 70	CG + hip extension-flexion and abduction–adduction exercises, knee extension-flexion exercises, ankle dorsiflexion-plantarflexion exercises	Physiotherapy, regular exercises, and weight-bearing exercises: up and down stairs, variable speed walking, and walking fast and stopping fast	TUG	13 (90 days)	7	30	47

*EG* experimental group, *CG* control group, *BMI* body mass index, *ROM* range of motion, *6MWT* six-minute walk test, *TUG* timed-up and go.

### Risk of bias in the included studies

As shown in Table 2, six studies showed a low overall risk of bias. Eight studies had one or two domains with “some concerns,” so their overall risk was rated as “some concerns.” None of the studies had a high risk of bias. For each domain, bias mainly arose from an implicit description of the randomization process (domain 1, 8 in 14 studies). Two studies had some concerns with domain 2, one with domain 3, and three with domain 5. All studies had a low risk of bias with domain 4 (bias in the measurement of the outcome).

**Table 2:** Risk of bias in the included studies.

Study	D1	D2	D3	D4	D5	Overall
Moffet	L	L	L	L	L	L
Jakobsen	L	L	L	L	L	L
Vuorenmaa	L	L	L	L	L	L
Bily	SC	SC	L	L	L	SC
Harikesavan	SC	L	L	L	L	SC
Jørgensen	SC	L	SC	L	L	SC
Husby	SC	L	L	L	L	SC
Schache	L	L	L	L	L	L
Do	L	L	L	L	L	L
Huang	SC	L	L	L	SC	SC
Liao	L	L	L	L	L	L
Trudelle	SC	SC	L	L	L	SC
Quan	SC	L	L	L	SC	SC
Sun	SC	L	L	L	SC	SC

*L* low risk of bias, *SC* some concerns.

### Meta-analyses results

#### 6MWT

**Figure 2:**
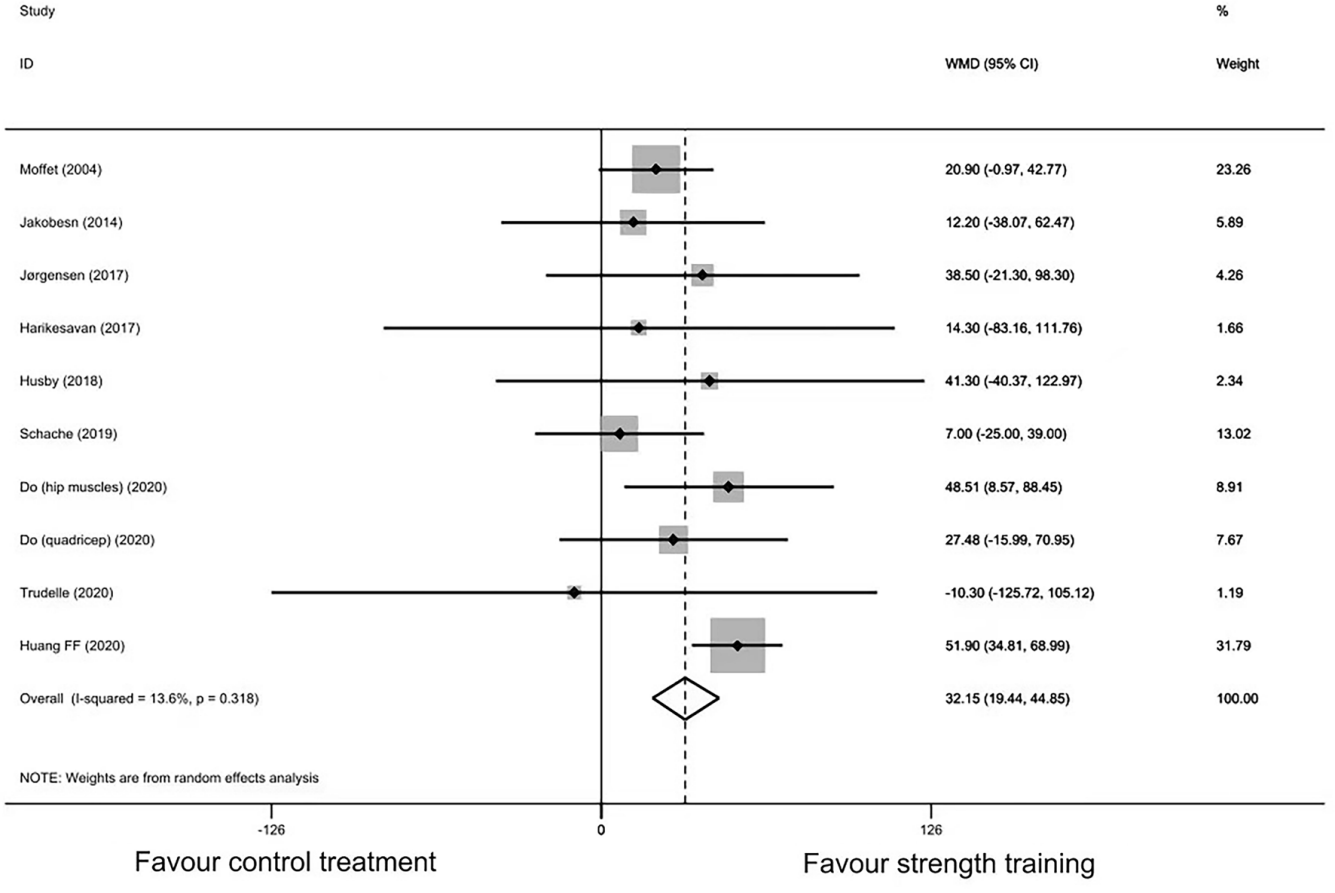
Forest plot of randomized controlled trails of strength training vs. control treatment: six-minute walk test.

Ten studies encompassing 535 participants had available data regarding 6MWT. The point estimates of the included studies were on the same side, except for the study by Trudelle et al.^42^ (including only 12 participants), which showed a 10.3 m smaller increase in 6MWT distance in the strength training group compared with the control group. Random-effect meta-analysis showed statistically significant between-group differences (MD: 32.15, 95% CI 19.44–44.85, *P* < 0.001, I^2^ = 13.6%) (Fig. 2). However, out of the 10 included studies, only the studies by Huang et al. and Do et al. showed that strength training could improve walking distance relative to the control group with statistical significance. Sensitivity analysis showed that the study by Huang et al. might be influential (see Supplementary Fig. S2 and Table S3 online), but none of its eligibility criteria, baseline characteristics of participants, and treatment protocols in both groups were outlined after reassessing. Besides, the overall point estimate after leaving out Huang’s study was still within the 95% CIs. Thus, that study was still included in the meta-regression. The funnel plot was asymmetric, but the Egger test did not show any potential publication bias (*p* = 0.19) (see Supplementary Fig. S4 online). The MD within the strength training group from baseline to posttreatment was 112.4 (95% CI 38.6–186.2) meters (see Supplementary Fig. S5 online).

#### TUG

**Figure 3:**
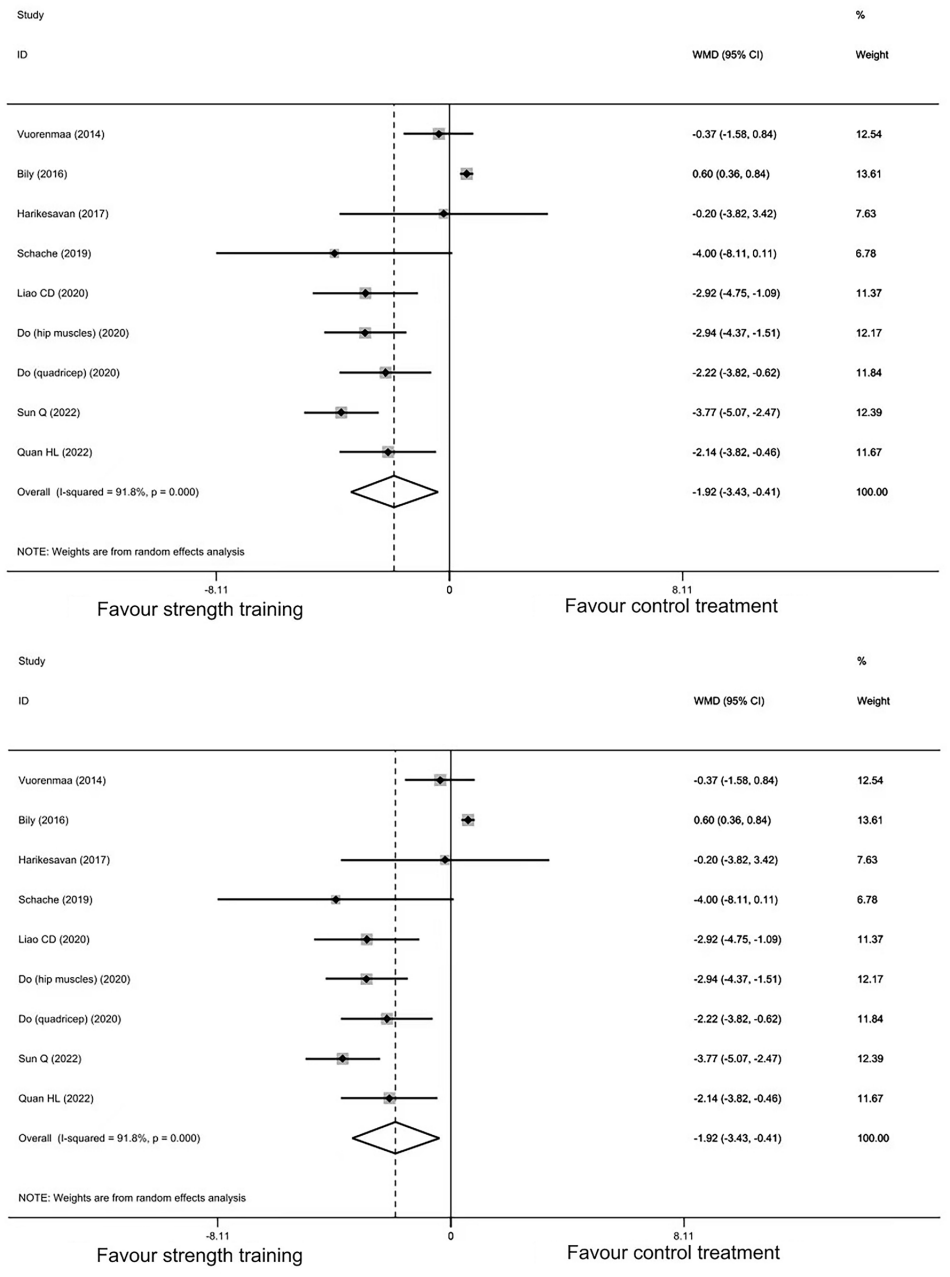
Forest plot of randomized controlled trails of strength training vs. control treatment: timed-up and go.

Eight studies encompassing 601 participants presented data for TUG. The point estimates of the included studies were on the same side, except for the study by Bily et al.^43^, which showed that there was a significant 0.6 s lower decrease in time to complete TUG in the strength training group than in the control group. Random-effect meta-analysis showed statistically significant between-group differences (MD: − 1.92, 95% CI − 3.43 to − 0.41, *P* = 0.012) (Fig. 3). The value of I^2^ was 92%, which indicated that there might be heterogeneity between the studies. Sensitivity analysis did not show obvious changes in the effect estimates by the leave-one-out method (see Supplementary Fig. S6 and Table S7 online). The MD within the strength training group from baseline to posttreatment was − 5.4 (95% CI − 7.6 to − 3.1) seconds (see Supplementary Fig. S8 online).

### Confidence level of meta-analyses results

The confidence level of our meta-analyses results was evaluated by the GRADE tool. For 6MWT, the confidence level was downgraded from high to moderate due to inconsistency (moderate-quality evidence), and for TUG, the confidence level was downgraded from high to low due to imprecision and inconsistency. The reasons for downgrading are shown in Supplementary Figs. S9 and S10 online.

### Meta-regression results and dose–response relationship

A univariate meta-regression was performed for the four strength training parameters (duration, frequency, volume, and initial time) separately.

### 6MWT (10 studies, 535 participants)

Regarding the dose–response relationship, volume showed a decreasing trend in 6MWT when training time increased between 15 and 70 min per session (*P* = 0.019, coefficient =  − 0.91, 95% CI − 1.63 to − 0.20). With no statistical significance, initial time showed a decreasing trend in 6MWT when strength training was initiated from 2 to 423 days after surgery (*P* = 0.85, coefficient =  − 0.02, 95% CI − 0.24–0.20). There was an increasing trend in 6MWT when training weeks increased from 7 to 12 (*P* = 0.18, coefficient = 5.04, 95% CI − 2.93–13.02) and training frequency increased from 1.5 (average) to 7 times per week (*P* = 0.34, coefficient = 3.30, 95% CI − 4.16–10.76) (see Supplementary Figs. S11–S14).

### TUG (9 studies, 601 participants)

We did not find dose–response relationships between the four strength training parameters and TUG. There was a decreasing trend in time to complete TUG when training weeks increased from 4 to 16 (*P* = 0.56, coefficient =  − 0.10, 95% CI − 0.48–0.29), training frequency increased from 2 to 7 times per week (*P* = 0.08, coefficient =  − 0.59, 95% CI − 1.28–0.10), training time increased between 15 and 60 min per session (*P* = 0.58, coefficient =  − 0.004, 95% CI − 0.027–0.018), and strength training was initiated from 8 to 180 days after surgery (*P* = 0.68, coefficient =  − 0.017, 95% CI − 0.23–0.20) (see Supplementary Figs. S15–S18).

## Discussion

In this dose–response meta-analysis, the general effects of strength training on functional ambulation following KR were first evaluated. Then, we explored the dose–response relationships between the four predetermined training parameters and the outcome, which was a critical aspect of rehabilitation following KR.

### Effects of strength training on functional ambulation after KR

Our study found a larger improvement (6MWT: 32 m, TUG: − 1.9 s) in functional ambulation of the strength training group relative to the control group. As a long-term goal after KR, functional ambulation improvement involves demands for primary goals such as muscle strength restoration, balance control, and pain management^6,50,51^. Hence, our results could, from another point of view, confirm the results of previous studies that adding strength training to rehabilitation protocols after KR could improve these primary goals^24,26,39^. Moreover, this finding could complement the APTA guideline on the benefits of strength training after KR to some extent, in addition to improvement of muscle strength, mobility, balance, and knee extension^10^.

We also computed the differences between baseline and posttreatment within the strength training group. The results showed 112.4 m increase for 6MWT (the study by Jakobsen et al.^40^ was not included because its baseline was preoperative) and − 5.4 s decrease for TUG, both of which reached the minimal detectable change (61.34 m at a 90% confidence level^52^ and − 2.27 s at a 95% confidence level^53^, respectively). Potential mechanisms of functional ambulation improvement following strength training might be explained by neuromuscular reactivation. Patients following KR may exhibit quadricep weakness and atrophy resulting from long-term KOA and the surgery, which can further lead to persistent neuromuscular activation deficits^6,54^. Such deficits can limit the ability to generate force and result in poor physical functions^6^. Strength training could cause a series of neurological reactivation at cortical or spinal level, including reduced variability of motor unit discharge rate, decreased motor unit recruitment threshold, increased motor neuron output, and enhanced muscle strength^55–58^.

### Dose–response relationship between strength training and functional ambulation

### Volume

Meta-regression suggested that there was a dose–response relationship between volume and 6MWT result with a decreasing trend. Such results differed both from our hypothesis that the training effect would increase, at least to a certain range, with the increase in training volume, and a previous study that showed similar increases in muscle strength among small and high volumes of strength training^59^. A far-fetched explanation could be that a large training volume could cause pain and muscle fatigue, thereby reducing performance in functional ambulation. However, considering parameters separately was likely to neglect the interactions among them, as basic information for a strength training prescription should contain intensity, frequency, and repetitions in addition to volume^60^.

### Frequency and duration

Some trends among the training parameters and outcomes were found, though there were no significant dose–response relationships. Improvements were found in both 6MWT (increased meters) and TUG (decreased seconds) with the increases in training duration and frequency. We were not able to explore the combined contributions of these two parameters to the outcome as neither of them had a dose–response relationship. Some previous studies have suggested that total training time rather than single frequency or duration improves strength^19^. When patients are trained one to three times per week and the total training time is unequaled, the muscle force increase is related to a long duration with higher frequency rather than frequency itself^20^. When the training frequency is three times per week, a longer total training time results in a greater muscle force increase^21^.

### Initial time

A slight decreasing trend of improvement was observed in 6MWT with postponed initial time (ranging from 0 to 423 days after surgery), meaning that early strength training has a higher potential benefit than late strength training. Current evidence suggests that early training, also known as fast-track rehabilitation, can shorten the in-patient stage and alleviate disfunction^61,62^. Neuromuscular activation deficits would persist until training and cause muscle weakness and immobility^54,63,64^. Early strength training activates neuromuscular control shortly after surgery, leading to an increase of 147% and 112% in muscle force and walking speed, respectively, in 2 weeks^22^ and preventing further muscle atrophy^58,65^.

A decreasing trend was observed in TUG, suggesting that early strength training had smaller potential benefits than late strength training. This seems to disagree with much of the existing research (mentioned in the last paragraph). The reasons may be that those studies found that earlier strength training was associated with better strength improvement rather than functional ambulation directly.

## Limitations and conclusions

There are several limitations to this study: (1) Due to ethical concerns, it is difficult to explore the isolated dose–response relationship between strength training and functional ambulation. We defined training protocols as strength training protocols if time of strengthening exercises constituted more than half of the total training time. Potential selection bias might exist due to subjective judgment of these protocols, though the study selection process was done by two independent reviewers, and discrepancies were finally resolved. (2) Previous studies have confirmed that strength training is not inferior to standard rehabilitation for the improvement of strength and functional ambulation^8,9^. Our results further showed that strength training could improve 6MWT relative to standard care or no treatment. However, only two out of 10 studies showed statistically significant results. Thus, the results of our study should be interpreted with caution, and more studies are warranted for a robust conclusion. (3) Studies in healthy adults have shown that training intensity is a critical parameter for the improvement of muscle strength and functional ability^66,67^. However, insufficient data for this parameter were presented in six included studies, so we were not able to explore the dose–response relationship between it and functional ambulation. In addition, progression is another important parameter during strength training that may affect the outcome, but it is difficult to compare the effects of various progressive designs through meta-regression. The results of this review only represent the mean effect of a period of strength training on functional ambulation in patients after KR. (4) The I^2^ statistic test for evaluating heterogeneity in our study depends on sample size but we did not further explore this issue with other tools (e.g., the prediction interval)^68^. (5) We did not search databases for grey literatures (e.g., Google Scholar), which might lead to potential bias in the searching process according to the Risk of Bias in Systematic Reviews (ROBIS) criteria^69^.

In conclusion, though moderate-certainty evidence suggests that strength training could increase 6MWT distance, the result should be interpreted with caution, as only two out of 10 studies showed statistically significant results. Moreover, low-certainty evidence shows that strength training could decrease time to complete TUG after KR. A dose–response relationship was only found between volume and 6MWT (a decreasing trend), which is far from the complete picture of relationships between strength training and functional ambulation after KR. More studies with explicit reports of strength training parameters are needed for further exploration of this topic.

## Supplementary Information

Supplementary Information.

## Data Availability

All data generated or analyzed during this study are included in this published article and its supplementary information files.
