# Supplementary material for: Effects of strength training on functional ambulation following knee replacement: a systematic review, meta-analysis, and meta-regression
Source: Sci Rep. 2023 Jul 3;13:10728. doi: 10.1038/s41598-023-37924-1 (PMC10317962; doi:10.1038/s41598-023-37924-1)
Supplement: Supplementary file 1 — Supplementary Information. [file 41598_2023_37924_MOESM1_ESM.pdf]

Effects of strength training on functional ambulation following knee replacement: A systematic review, meta-analysis, and meta-regression

Jinxiang Wang[1,2], Ranran Zhu[2], Xiao-ting Xu[3], Shuting Liu[2], Zhenrui Li[1], Chaoyang Guo[2], Xuchen Tao[2], Liang Qian[2], Ravon Charles[2], *Lei Fang[1,2]

[1]Yueyang Hospital of Integrated Traditional Chinese and Western Medicine, Shanghai University of Traditional Chinese Medicine, Shanghai, China

[2]School of Rehabilitation Science, Shanghai University of Traditional Chinese Medicine, Shanghai, China

[3]Institute of TCM International Standardization, Shuguang Hospital Affiliated to Shanghai University of Traditional Chinese Medicine, China

*Correspondence to: Lei Fang, email: fanglei586@126.com

Supplementary Table S1 Full search strategy

Database	Strategy	Outcome
Pubmed	((((arthroplasty, knee replacement[MeSH Terms]) OR (knee replacement[Title/Abstract])) OR (knee arthroplasty[Title/Abstract])) OR (Knee Prosthesis[Title/Abstract])) AND (((((((Resistance Training[MeSH Terms]) OR (Strength Training[Title/Abstract])) OR (Weight-Bearing Exercise[Title/Abstract])) OR (eccentric exercise[Title/Abstract])) OR (concentric exercise[Title/Abstract])) OR (isotonic exercise[Title/Abstract])) OR (Weight Lifting Exercise[Title/Abstract]))	541
Web of Science	(arthroplasty, knee replacement OR knee replacement OR knee arthroplasty OR Knee Prosthesis) AND (Resistance Training OR Strength Training OR Weight-Bearing Exercise OR eccentric exercise OR concentric exercise OR isotonic exercise OR Weight Lifting Exercise)	898
Cochrane	(arthroplasty, knee replacement OR knee replacement OR knee arthroplasty OR Knee Prosthesis) AND (Resistance Training OR Strength Training OR Weight-Bearing Exercise OR eccentric exercise OR concentric exercise OR isotonic exercise OR Weight Lifting Exercise) in Title Abstract Keyword	1684
Ovid	(arthroplasty, knee replacement OR knee replacement OR knee arthroplasty OR Knee Prosthesis) AND (Resistance Training OR Strength Training OR Weight-Bearing Exercise OR eccentric exercise OR concentric exercise OR isotonic exercise OR Weight Lifting Exercise) ｛Including Limited Related Terms｝	3451
Embase	(arthroplasty, knee replacement OR knee replacement OR knee arthroplasty OR Knee Prosthesis) AND (Resistance Training OR Strength Training OR Weight-Bearing Exercise OR eccentric exercise OR concentric exercise OR isotonic exercise OR Weight Lifting Exercise) ti,ab,kw AND “randomized controlled trail”/de	586
CNKI	“膝关节置换” AND（“力量” OR “抗阻” OR “渐进性”）	138

WANFANG DATA	“膝关节置换” AND（“力量” OR “抗阻” OR “渐进性”）	102
VIP	“膝关节置换” AND（“力量” OR “抗阻” OR “渐进性”）	48

Supplementary Fig. S2 Sensitivity analysis of 6MWT

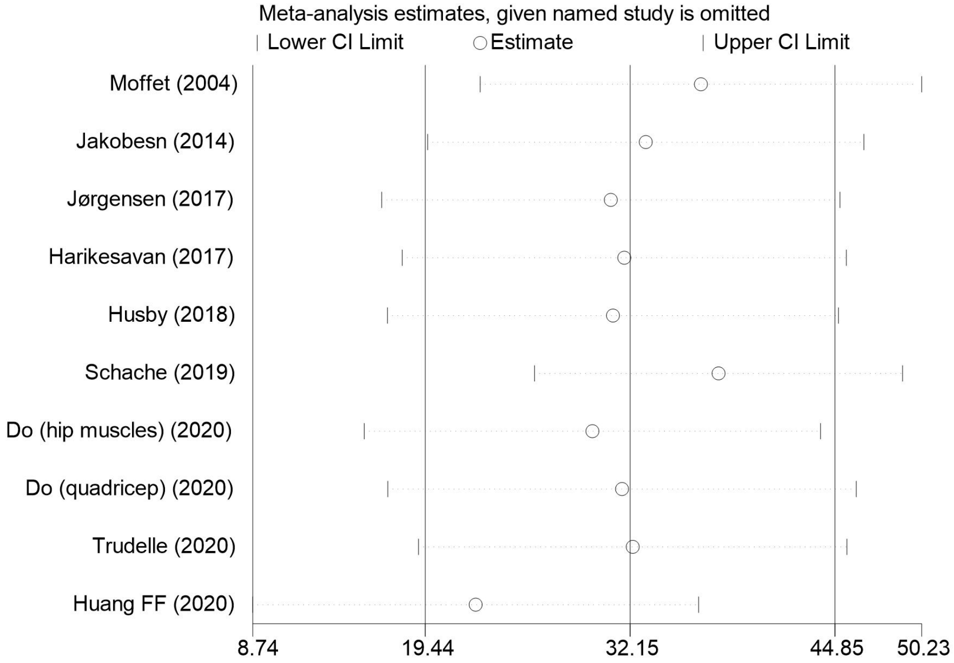

Supplementary Table S3 Sensitivity analysis of 6MWT

Study omitted	Estimate	lower 95% CI	upper 95% CI
Moffet (2004)	36.54	22.85	50.23
Jakobesn (2014)	33.12	19.59	46.65
Jørgensen (2017)	30.95	16.74	45.16
Harikesavan (2017)	31.79	18.01	45.56
Husby (2018)	31.08	17.10	45.07
Schache (2019)	37.63	26.22	49.04
Do (hip muscles) (2020)	29.81	15.65	43.96
Do (quadricep) (2020)	31.65	17.12	46.17
Trudelle (2020)	32.31	19.02	45.60
Huang FF (2020)	22.57	8.74	36.40
Combined	32.15	19.44	44.85

Supplementary Fig. S4 Funnel plot of 6MWT

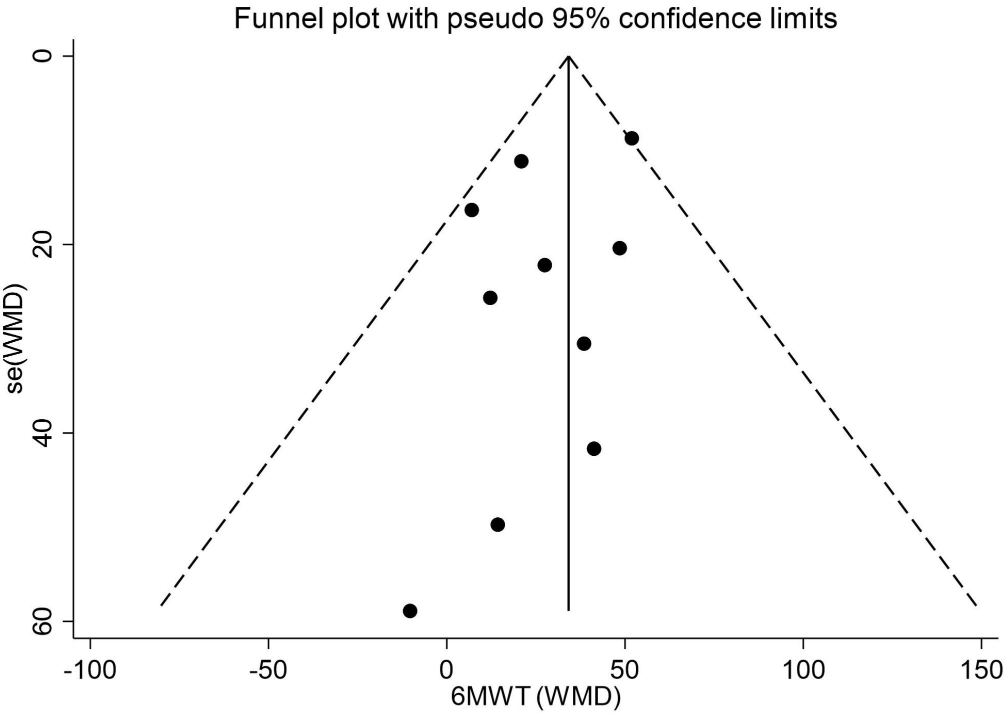

Supplementary Fig. S5 Within group mean difference for strength training (6MWT)

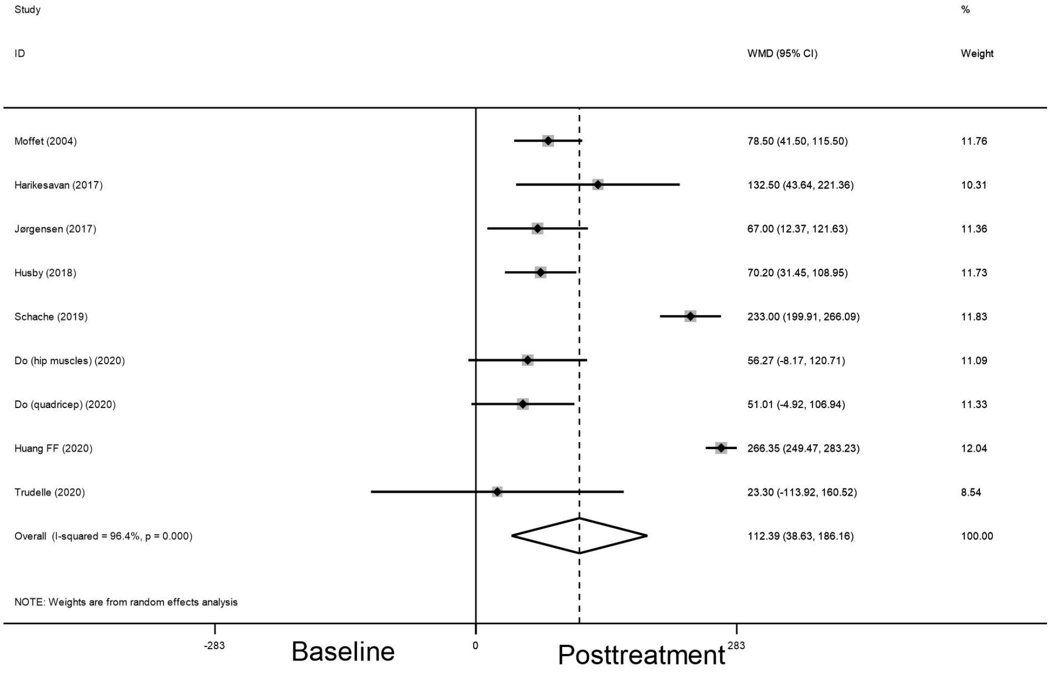

Supplementary Fig. S6 Sensitivity analysis of TUG

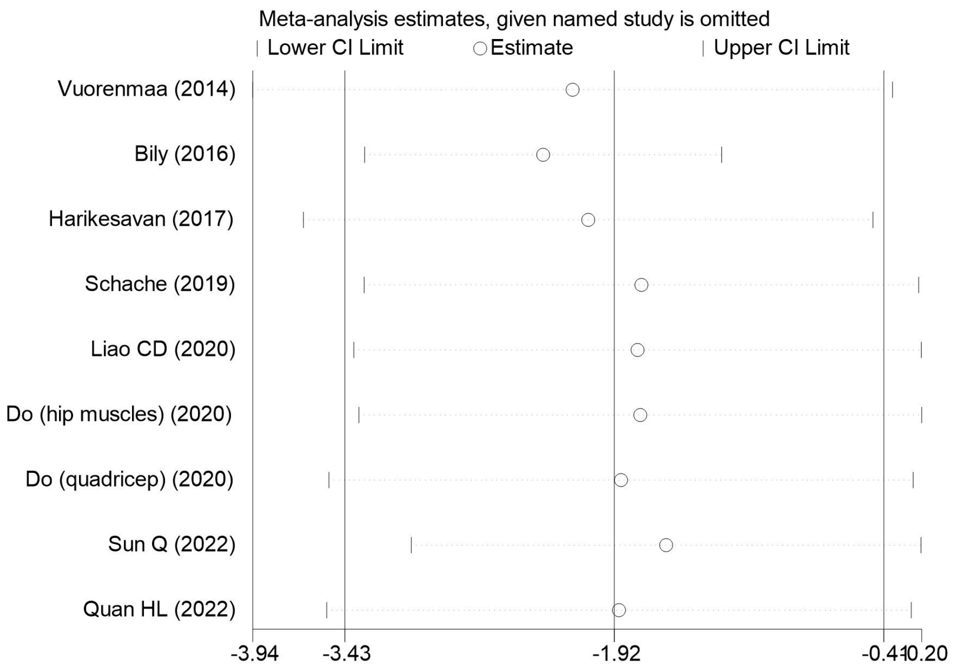

Supplementary Table S7 Sensitivity analysis of TUG

Study omitted	Estimate	lower 95% CI	upper 95% CI
Vuorenmaa (2014)	-2.15	-3.94	-0.37
Bily (2016)	-2.32	-3.31	-1.32
Harikesavan (2017)	-2.07	-3.66	-0.48
Schache (2019)	-1.77	-3.32	-0.22
Liao CD (2020)	-1.79	-3.38	-0.21
Do (hip muscles) (2020)	-1.78	-3.35	-0.20
Do (quadricep) (2020)	-1.88	-3.52	-0.25
Sun Q (2022)	-1.63	-3.05	-0.21
Quan HL (2022)	-1.89	-3.53	-0.26
Combined	-1.92	-3.43	-0.41

Supplementary Fig. S8 Within group mean difference for strength training (TUG)

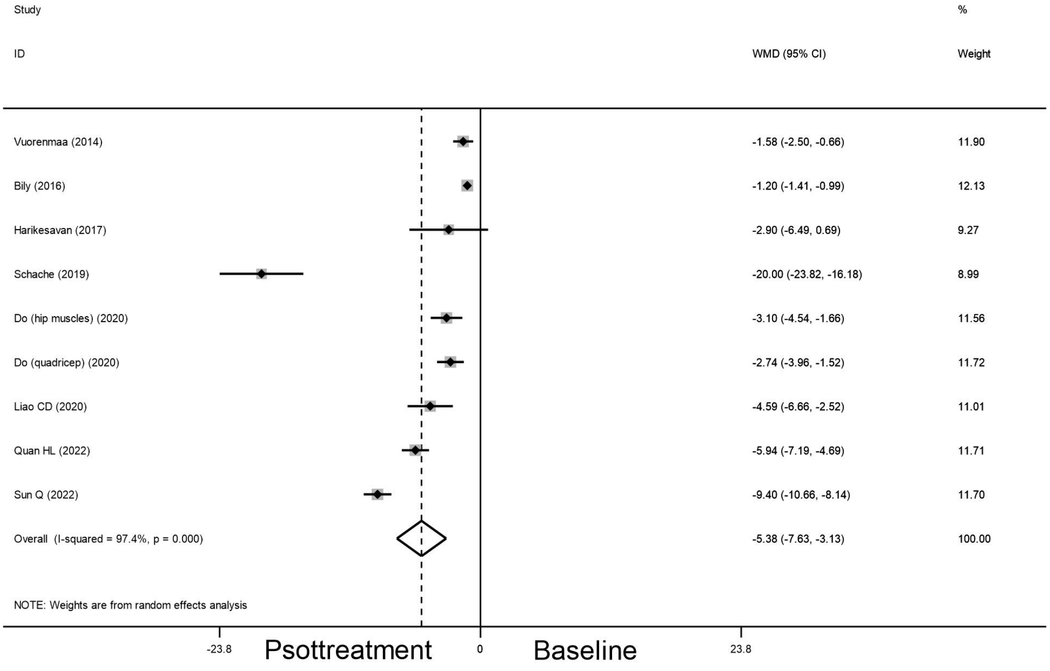

Supplementary Fig. S9 Certainty assessment (6MWT)

Strength training vs control treatment

Bibliography:

Outcomes	No of Participants (studies) Follow up	Quality of the evidence (GRADE)	Relative effect (95% CI)	Anticipated absolute effects: Risk with Control 6MWT	Anticipated absolute effects: Risk difference with Strength trainig (95% CI)
**6MWT**	535 (10 studies)	⊕⊕⊕⊝ **MODERATE**[1] due to imprecision			The mean 6mwt in the intervention groups was **32.15 higher** (19.44 to 44.85 higher)

*The basis for the **assumed risk** (e.g. the median control group risk across studies) is provided in footnotes. The **corresponding risk** (and its 95% confidence interval) is based on the assumed risk in the comparison group and the **relative effect** of the intervention (and its 95% CI).

CI: Confidence interval;

GRADE Working Group grades of evidence
High quality: Further research is very unlikely to change our confidence in the estimate of effect.
Moderate quality: Further research is likely to have an important impact on our confidence in the estimate of effect and may change the estimate.
Low quality: Further research is very likely to have an important impact on our confidence in the estimate of effect and is likely to change the estimate.
Very low quality: We are very uncertain about the estimate.

[1] wide 95% CI

Supplementary Fig. S10 Certainty assessment (TUG)

Strength training vs control treatment

Bibliography:

Outcomes	No of Participants (studies) Follow up	Quality of the evidence (GRADE)	Relative effect (95% CI)	Anticipated absolute effects: Risk with Control	Anticipated absolute effects: Risk difference with TUG (95% CI)
**TUG**	601 (9 studies)	⊕⊕⊝⊝ **LOW**[1,2] due to inconsistency, imprecision			The mean tug in the intervention groups was **1.92 lower** (3.43 to 0.41 lower)

*The basis for the **assumed risk** (e.g. the median control group risk across studies) is provided in footnotes. The **corresponding risk** (and its 95% confidence interval) is based on the assumed risk in the comparison group and the **relative effect** of the intervention (and its 95% CI).

CI: Confidence interval;

GRADE Working Group grades of evidence
High quality: Further research is very unlikely to change our confidence in the estimate of effect.
Moderate quality: Further research is likely to have an important impact on our confidence in the estimate of effect and may change the estimate.
Low quality: Further research is very likely to have an important impact on our confidence in the estimate of effect and is likely to change the estimate.
Very low quality: We are very uncertain about the estimate.

[1] I2 > 50%
[2] wide 95% CI

domain for downgrading:
risk of bias: over 50% studies were high risk of bias
inconsistency: I^2 over 50%
indirectness: studies included or meta-analysis approaches were irrelevant with the study aim
imprecision: wide 95% confidence interval (cover the minimal important difference)
publication bias: if publication exists

Supplementary Fig. S11 Dose-response relationship between strength training duration and 6MWT

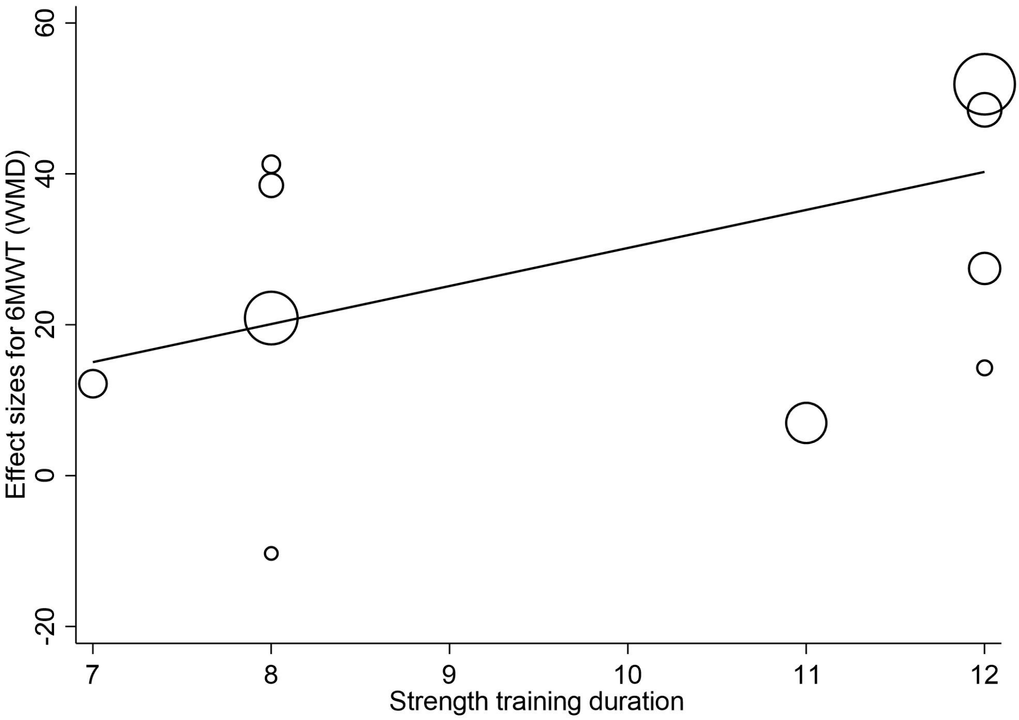

Supplementary Fig. S12 Dose-response relationship between strength training frequency and 6MWT

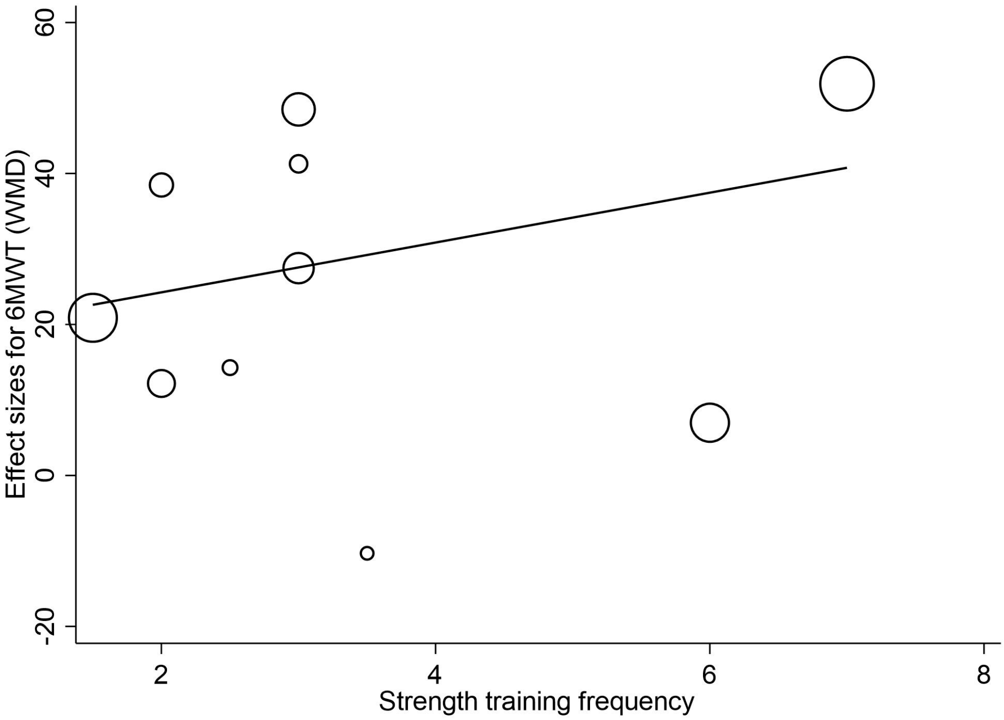

Supplementary Fig. S13 Dose-response relationship between strength training volume and 6MWT

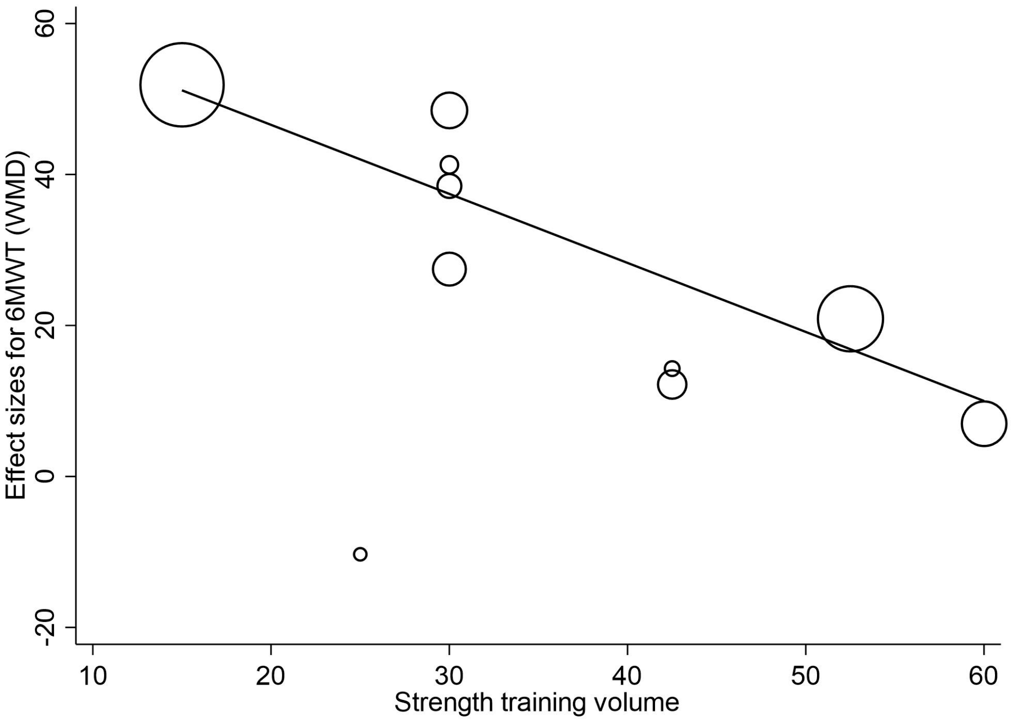

Supplementary Fig. S14 Dose-response relationship between strength training initial time and 6MWT

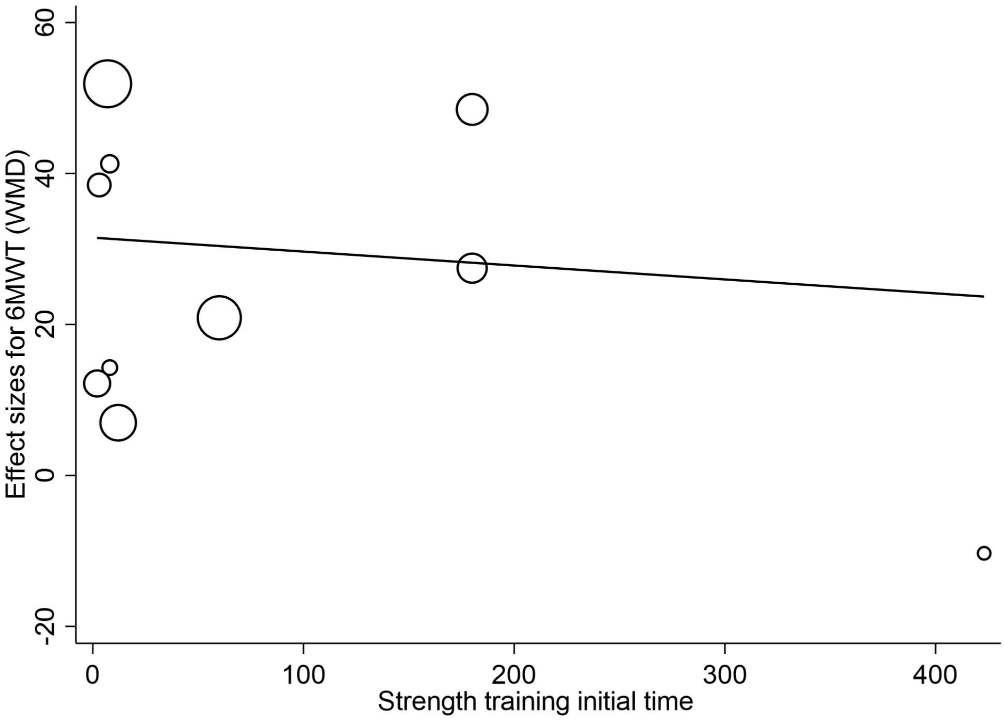

Supplementary Fig. S15 Dose-response relationship between strength training duration and TUG

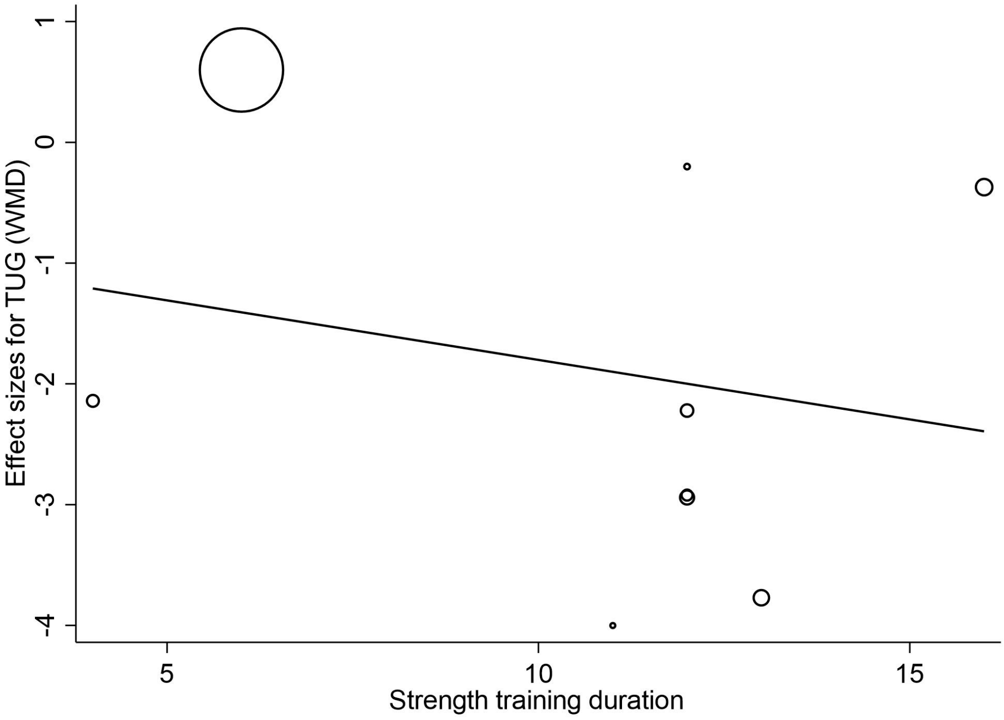

Supplementary Fig. S16 Dose-response relationship between strength training frequency and TUG

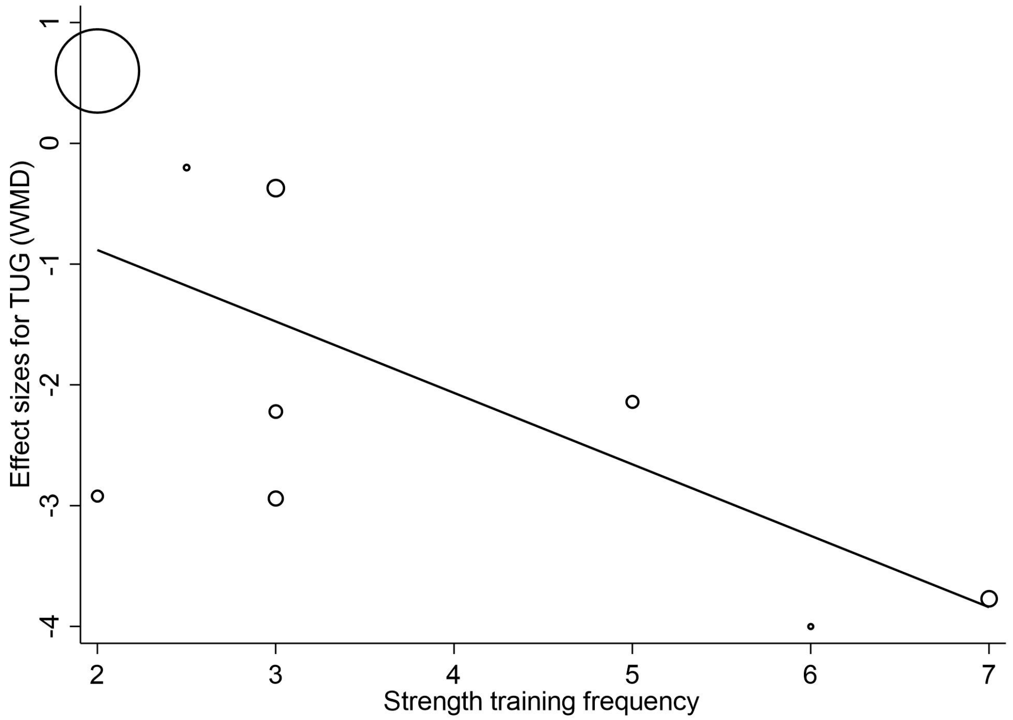

Supplementary Fig. S17 Dose-response relationship between strength training volume and TUG

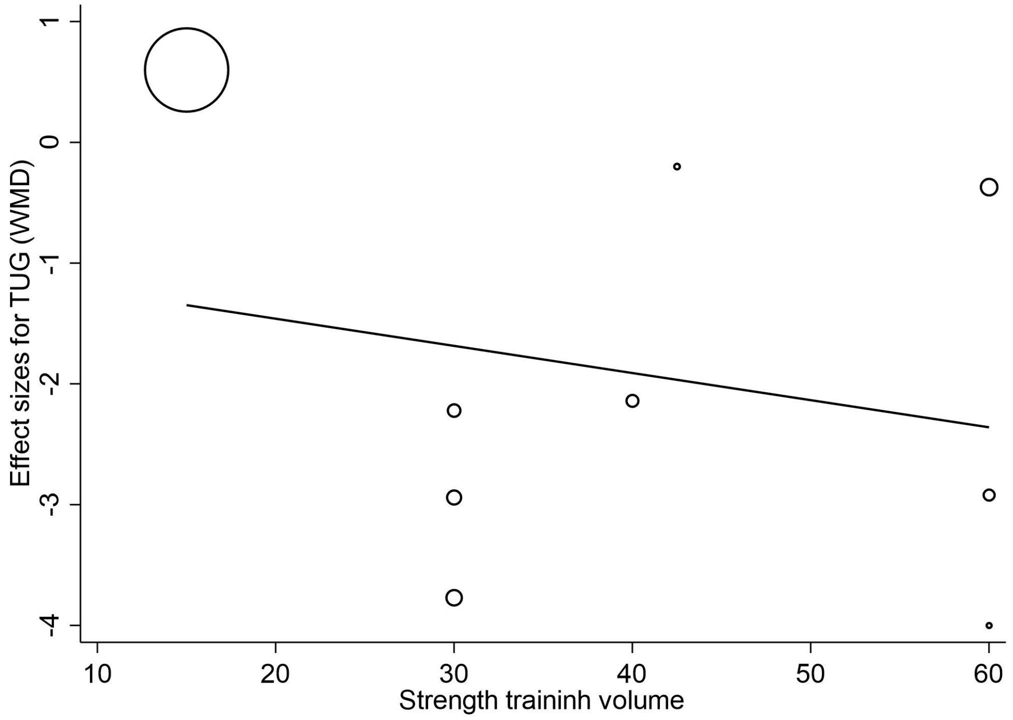

Supplementary Fig. S18 Dose-response relationship between strength training initial time and TUG

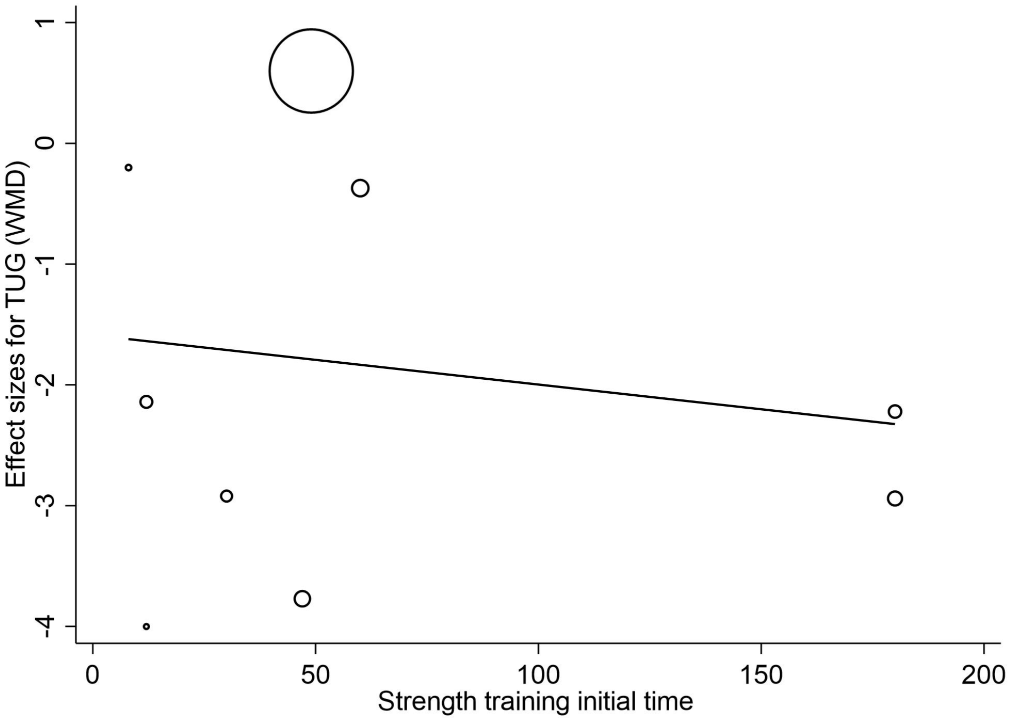
Effect sizes for TUG (WMD)
1
0
-1
-2
-3
-4
0
50
100
150
200
Strength training initial time
